# Kocuria rhizophila Infection: A Case Report and Literature Review

**DOI:** 10.7759/cureus.86788

**Published:** 2025-06-26

**Authors:** Raquel Azevedo, Ana Luísa Graça, Glória Gonçalves, Ana Cláudia Carvalho

**Affiliations:** 1 Infectious Diseases, Unidade Local de Saúde de Braga, Braga, PRT; 2 Pharmacology, Unidade Local de Saúde de Braga, Braga, PRT

**Keywords:** bloodstream infections, device-related infections, emergent pathogen, kocuria rizophila, opportunistic bacteria

## Abstract

The *Kocuria *species, formerly considered opportunistic bacteria, have been described as capable of producing infection in various systems and organs, particularly among immunocompromised patients, and are associated with device-related infections.

We report a 54-year-old immunocompetent woman with relapsing central venous catheter-related infection following a seven-day course of amoxicillin and clavulanic acid. Blood cultures and catheter tips grew *Kocuria rhizophila*. Susceptibility testing was performed and interpreted according to *Staphylococcus* species criteria, showing sensitivity to all tested antibiotics. Catheter removal and antibiotic treatment resulted in a complete resolution.

To our knowledge, we present the eighth case report of infection by *Kocuria rhizophila*. Infections caused by this pathogen are usually observed in patients with a history of immunosuppression or vascular catheters. Consequently, the occurrence of *Kocuria* species in this context demands clinical awareness. Further studies and detailed reporting of clinical cases are essential to improve diagnostic accuracy, guide treatment decisions, and clarify the role of *Kocuria* spp. as an emerging pathogen.

## Introduction

*Kocuria* species, formerly considered to be only bacterial commensals of the oropharyngeal mucosa and skin, have now emerged as human pathogens. Thus far, five species have been described as pathogenic for humans: *K. rhizophila*, *K. kristinae*, *K. rosae*, *K. marina*, and *K. varians *[1].

Organisms in the genus *Kocuria *are Gram-positive, catalase-positive, and coagulase-negative coccoid bacteria that belong to the family Micrococcaceae, order Actinomycetales, and class Actinobacteria [2].

In the past, microbiological identification techniques were unable to accurately identify *Kocuria *species, resulting in an underestimation of the prevalence of *Kocuria*-related infections. Moreover, the increasing complexity of patients makes them more susceptible to less virulent organisms. As a result, the number of reported *Kocuria* infections has increased with recent advancements in surveillance technology, especially among immunocompromised individuals and in device-related illnesses [3-5]. For these reasons, although disease caused by this pathogen remains extremely uncommon, the isolation of *Kocuria* spp. in clinical samples should be carefully correlated with the clinical condition rather than being immediately dismissed as contamination [3].

To our knowledge, this is the eighth case report of infection by *Kocuria *described worldwide, and one of the few in immunocompetent patients.

This case report was previously presented as a meeting abstract at the “Encontro sobre Infeção Hospitalar - ULS Braga,” on April 8, 2024.

## Case presentation

We report a 54-year-old woman with a history of hypothyroidism refractory to oral treatment, managed with weekly levothyroxine via a totally implantable central venous catheter (CVC) since 2019.

In December 2022, the patient presented with localized erythema and tenderness over the CVC port site, without purulent discharge. She was prescribed amoxicillin and clavulanic acid (875 mg + 125 mg), twice a day for seven days, and temporarily discontinued administration of levothyroxine via the CVC, leading to symptom resolution.

One month later, the patient presented with intermittent fever, coinciding with the weekly intravenous medication administration through the CVC. On examination, her temperature was 37.5ºC, blood pressure 130/80 mmHg, pulse 96/min, and respiratory rate 16/min. At this point, no signs of local inflammation were present. Laboratory data showed a peripheral white blood cell (WBC) count of 9.2×10⁹/L and a C-reactive protein (CRP) level of 5.30 mg/L. Nonetheless, peripheral and CVC blood cultures were drawn. Although peripheral blood cultures were sterile, CVC blood cultures grew Gram-positive bacteria. The isolates were identified as *Kocuria rhizophila* using MALDI Biotyper® (Bruker Daltonics, Bremen, Germany), a mass spectrometry-based technique that identifies microorganisms by analyzing their unique protein spectra profiles against a reference database.

Considering the relapse of infection and prior episode of cellulitis over the port, the CVC was removed, and tip culture confirmed the presence of *Kocuria rhizophila*. Accounting for the previous good response, we prescribed amoxicillin and clavulanic acid (875 mg + 125 mg three times a day), pending susceptibility testing.

Susceptibility testing was performed using an agar disk diffusion assay. Based on the *Staphylococcus*-species interpretative criteria (Version 14.0 of EUCAST (European Committee on Antimicrobial Susceptibility Testing)), the *Kocuria rhizophila *strain isolated from our patient showed sensitivity to all antibiotics tested, namely ampicillin, ciprofloxacin, clindamycin, erythromycin, and trimethoprim/sulfamethoxazole. Additionally, E-test® strip testing revealed a minimum inhibitory concentration (MIC) of <0.016 mg/L to penicillin, <0.016 mg/L to amoxicillin-clavulanic acid, and 0.5 μg/mL to vancomycin (interpretation according to *Staphylococcus*-species susceptibility criteria: susceptible). Susceptibility testing results are summarized in Table 1.

**Table 1:** Antimicrobial susceptibility results of Kocuria rhizophila isolated Antimicrobial susceptibility results of *Kocuria rhizophila* isolated from CVC blood cultures. Interpretations are based on EUCAST v14.0 breakpoints for *Staphylococcus spp.* EUCAST*, *European Committee on Antimicrobial Susceptibility Testing.

Antibiotic	Method	Result	Interpretation
Ampicillin	Disk diffusion	Sensitive	Susceptible
Ciprofloxacin	Disk diffusion	Sensitive	Susceptible
Clindamycin	Disk diffusion	Sensitive	Susceptible
Erythromycin	Disk diffusion	Sensitive	Susceptible
Trimethoprim/sulfamethoxazole	Disk diffusion	Sensitive	Susceptible
Penicillin	E-test®	MIC < 0.016 mg/L	Susceptible
Amoxicillin-clavulanic acid	E-test®	MIC < 0.016 mg/L	Susceptible
Vancomycin	E-test®	MIC 0.5 µg/mL	Susceptible

The patient’s fever resolved, and follow-up blood cultures were sterile. She completed a seven-day course of treatment. A new CVC was inserted nine days after the removal of the infected catheter. The patient was discharged from the infectious diseases clinic after resolution of the infection episode and continued regular follow-up in the endocrinology clinic and the medical day hospital. No recurrence of CVC infection caused by the same agent was documented over a two-year period. The clinical course, microbiological findings, and management decisions are outlined in Table 2.

**Table 2:** Clinical course and management timeline Summary of clinical course, microbiological findings, antimicrobial therapy, and clinical management. BID, twice a day; TID, three times a day; CVC, central venous catheter.

Timepoint	Event
2019	Initiation of weekly intravenous levothyroxine via totally implantable CVC
December 2022	Local erythema and tenderness over CVC port site; treated with amoxicillin-clavulanic acid (875/125 mg BID for 7 days); levothyroxine via CVC temporarily withheld
January 2023	Intermittent fever after CVC use; peripheral blood cultures negative, CVC cultures positive for* K. rhizophila*
Upon diagnosis	CVC removed; tip culture confirmed *K. rhizophila *presence
Treatment phase	Amoxicillin-clavulanic acid (875/125 mg TID) for 7 days
9 days post CVC removal	New CVC inserted
Post-resolution follow-up	Discharged from Infectious Diseases clinic; no recurrence over 2 years

## Discussion

*Kocuria *spp. compose the human flora and are most commonly treated as contaminants, which can contribute to the underdiagnosis of *Kocuria *spp. infections [6].

A recent literature review identified 102 cases of *Kocuria *spp. infection, mostly due to *K. kristinae *[7].

We focused on seven reports of infection by *K. rhizophila*, the first published in 2008 [8]. All were hardware-related infections: four CVC-related bloodstream infections [8-10], used for parenteral nutrition and one complicated by native valve endocarditis requiring surgery [11]; two cases of peritonitis (dialysis catheter-associated) [12,13]; and one prosthetic joint infection [14]. Patient age ranged from 3 to 81 years, with the extremes of age being particularly affected.

Different antibiotics were used, mostly β-lactams (cefotaxime, ceftriaxone, cefazolin, ceftazidime, vancomycin), in some cases combined with gentamycin. While some infections were effectively treated without device removal, recurrence also occurred. No fatalities were reported (Table 3).

**Table 3:** Summary of Kocuria rhizophila infections reported to date *Interpretation according to *Staphylococcus *spp. susceptibility criteria. ^#^DAIR procedure: debridement, antibiotics, and implant retention. ^ᵃ^Interpretation according to *Micrococcus *spp. susceptibility criteria. AMI, amikacin; AMO, amoxicillin; AMC, amoxicillin/clavulanic acid; BEN, benzylpenicillin; CZO, cefazolin; CTA, cefotaxime; CXI, cefoxitin; CIP, ciprofloxacin; CLI, clindamycin; ERY, erythromycin; GEN, gentamicin; KAN, kanamycin; LEV, levofloxacin; NOR, norfloxacin; OXA, oxacillin; TEI, teicoplanin; TET, tetracycline; TOB, tobramycin; TRS, trimethoprim/sulfamethoxazole; VAN, vancomycin; WBC, white blood cell; CRP, C-reactive protein.

Reference(s)/year of publication	Age (years)/sex	Underlying diseases or conditions	Focus of infection	Inflammatory parameters	Susceptibility pattern*			Treatment	Source control measures	Outcome
					S	I	R			
Becker et al. [8] /2008	8/Male	Methylmalonic aciduria under parenteral nutrition	Catheter-related bacteremia	Not reported	β-lactams, macrolides, glycopeptides, quinolones	—	NOR	Vancomycin (33 mg/kg/day, 7 days) + cefotaxime (100 mg/kg/day, 7 days)	Removal of the catheter	Cure
Moissenet et al. [9] /2012	3/Female	Hirschsprung's disease under parenteral nutrition	Catheter-related bacteremia	Not reported	BEN, GEN, AMI, TOB, TET, VAN, TEI	CF	ERY	Vancomycin (40 mg/kg/day, 10 days) + Gentamicin (3 mg/kg/day, 2 days)	Ethanol locks + catheter repair	Cure
Kim et al. [10] /2023	18/Male	Avoidant restrictive food intake disorder under parenteral nutrition	Catheter-related bacteremia	WBC: 3.58×10⁹/L; CRP: 26.1 mg/L	BEN^a^, ERY^a^, VAN^a^, GEN, OXA, TEI	CIP, LEV	—	Vancomycin (690 mg at six-hour interval, 16 days)	Vancomycin-lock therapy for 11 days	Cure
Pierron et al. [11] /2021	81/Male	Mechanical bowel obstruction under parenteral nutrition	Catheter-related bacteremia + native valve endocarditis	WBC: 15.5×10⁹/L; CRP: 110 mg/L	CXI, CAN, CLI, TRS, CTA	AMO, AMC, LEV	BEN, ERY, NOR	Cefotaxime (6 g/day) followed by ceftriaxone (2 g/day) for 6 weeks	Removal of catheter + aortic valvular replacement	Cure
Ishihara et al. [12] /2021	3/Female	Steroid-resistant nephrotic syndrome with end-stage renal disease on peritoneal dialysis	Catheter-related Peritonitis	WBC: 21.3×10⁹/L; CRP: 11.3 mg/L	Not reported			Intraperitoneal cefazolin and ceftazidime	Not reported	Cure
Nakata et al. [13] /2024	78/Male	End-stage kidney disease due to diabetic nephropathy on peritoneal dialysis	Catheter-related Peritonitis	WBC: 8.1×10⁹/L; CRP: 11.3 mg/L	Not reported			Intraperitoneal ceftazidime + vancomycin (7 days) followed by penicillin G (14 days)	Removal of the catheter	Cure
McAleese et al. [14] /2023	74/Male	Total hip replacement + polymyalgia rheumatica treated with methotrexate	Prosthetic joint infection	WBC: 8.5×10⁹/L; CRP: 14 mg/L	Not reported			Vancomycin + Piperacillin-Tazobactam followed by Vancomycin (12 weeks)	DAIR procedure#	Cure

Despite its rarity, the most common infections caused by *Kocuria *spp. are bacteremia, skin and soft tissue infection, endophthalmitis, infectious endocarditis, and peritonitis (with peritoneal dialysis identified as a risk factor). Infections by these pathogens are usually observed in patients with a history of malignancy, immunosuppression, or indwelling long-term catheters [7]. Studies have demonstrated that cancer patients present a higher risk of *Kocuria*-related infections, probably related to the immunosuppression associated with the malignancy and the treatment itself. Nevertheless, infections in immunocompetent individuals have also been described [15].

A common predisposing factor is the presence of a CVC, particularly if used for parenteral nutrition [7]. Indwelling catheters not only facilitate the entry of microorganisms into the bloodstream but also enable bacterial adherence. Biofilm development promotes bacterial growth and constitutes a mechanism of resistance to antibiotic treatment. Although there is no conclusive evidence that *Kocuria *spp. form biofilms, multiple studies indicate that catheter removal is determinant for complete infection resolution [3]. In our case, recurrence was perceived shortly after antibiotic discontinuation. Cellulitis over the hub in the first episode was interpreted as a sign of extraluminal infection. Since retention and antibiotic lock therapy are not indicated in such cases, CVC removal combined with effective antibiotic therapy was crucial for a definitive infection resolution.

Antibiotic treatment is not well defined. There are no treatment guidelines or susceptibility criteria for *Kocuria *spp., and most studies apply *Staphylococcus *breakpoints to interpret the microorganism’s behaviour under antimicrobial exposure [3]. A recent narrative review evaluated the antimicrobial resistance of *Kocuria *spp. infections and found the highest resistance associated with macrolides and penicillin. The lowest resistance rates were reported for vancomycin and tetracyclines; cephalosporin resistance was estimated at 18%. According to the same review, vancomycin was the most used antibiotic, followed by cephalosporins and quinolones. The duration of antimicrobial treatment remains uncertain and depends on the site of infection [7]. Of the seven cases of *Kocuria *rhizophila infection we reviewed, only four reported susceptibility testing [8-11]. Most strains showed resistance to antibiotics, namely to quinolones and erythromycin [8,9,11]. The publication of susceptibility testing results and clinical outcomes is essential for establishing clinical breakpoints. Based on the *Staphylococcus *species interpretative criteria, the *Kocuria rhizophila *strain isolated from our patient showed sensitivity to all antibiotics tested. Given our patient’s previous favourable clinical response to amoxicillin-clavulanic acid, the absence of severe infection signs, and CVC removal, we decided to prescribe the same regimen for a total of seven days.

Compared to previously reported cases of *Kocuria rhizophila *infection, which have predominantly involved immunocompromised individuals or patients receiving parenteral nutrition, our case is notable for occurring in an immunocompetent adult with a relatively mild course of disease. The resolution of symptoms following catheter removal suggests that device colonization and biofilm potential may be critical factors, regardless of host immunity status. This case adds to the limited clinical spectrum of *Kocuria *spp. infections and underscores the importance of host factors and device involvement in disease progression.

## Conclusions

Although infections caused by *Kocuria *spp. in immunocompetent individuals remain rare, this case contributes to growing awareness of its pathogenic potential. Accurate interpretation of *Kocuria *isolation requires careful clinical correlation, particularly in the presence of indwelling medical devices or recurrent isolation of the same organism. To avoid misdiagnosis or inappropriate treatment, clinicians should resist dismissing such findings as contamination without thorough assessment. Early consideration of device-related infection, coupled with timely removal and targeted antibiotic therapy, may prevent recurrence and unnecessary broad-spectrum antimicrobial use. Given the lack of standardized treatment guidelines, systematic follow-up, including repeat cultures and clinical monitoring, is essential to confirm resolution and guide management. Continued documentation of similar cases, especially those with susceptibility profiles and long-term outcomes, will be instrumental in shaping evidence-based recommendations and avoiding both under- and overtreatment.
